# Integrated Aquaponic Co‐Culture of Nile Tilapia and African Catfish: Effects on Plant Yield, Fish Growth, Nutrient Profile, and Heavy Metal Accumulation

**DOI:** 10.1002/fsn3.71333

**Published:** 2025-12-30

**Authors:** Jude Kifufu Gafidji Fidji, Réné Wamene Baranger, Gaétan Kalala Bolokango, Patrick Mafwila Kinkela, Honoré Kiatoko Mangeye

**Affiliations:** ^1^ Département de Zootechnie Université de Kinshasa, Faculté des Sciences Agronomiques et Environnement Kinshasa Democratic Republic of the Congo; ^2^ Institut Supérieur Pédagogique de Bandundu Mention Santé et Productions Animales Bandundu Democratic Republic of the Congo

**Keywords:** aquaponics, *Clarias gariepinus*, co‐culture, eggplant, *Oreochromis niloticus*

## Abstract

A 106‐day experiment was conducted to evaluate the co‐culture of Nile tilapia ( *Oreochromis niloticus* , 20.4 ± 0.29 g) and African catfish ( *Clarias gariepinus* , 19.8 ± 0.46 g) in an aquaponic system using eggplant ( *Solanum melongena* ) as vegetable specie. Fish were stocked at an initial biomass of 10 kg·m^−3^ with five different Nile tilapia: African catfish stocking ratios: 2:8 (TC_1_), 4:6 (TC_2_), 5:5 (TC_3_), 6:4 (TC_4_), and 8:2 (TC_5_), totalling 100 fish per tank. Results showed no significant differences (*p* > 0.05) in eggplant yield, Nile tilapia growth performance, or survival rate among treatments. However, African catfish growth performance declined with decreasing proportion of catfish, with a statistically significant difference observed only between the highest (TC_1_) and lowest (TC_5_) catfish ratios. The lowest survival rate (90.0%) was recorded in TC_5_. Proximate analysis revealed no significant variation (*p* > 0.05) in the nutritional composition of either fish species across treatments, and heavy metal levels in fish flesh remained below toxic thresholds. Water quality parameters remained within optimal ranges for both species and did not significantly influence fish growth. These findings support the feasibility of co‐culturing Nile tilapia and African catfish in aquaponic systems without compromising water quality, yield plant, fish performance, or nutritional value.

## Graphical abstract

An integrated aquaponic co‐culture system combining Nile tilapia and African catfish with eggplant was evaluated. Tilapia growth was consistent across treatments, while a slight reduction in catfish performance occurred with lower stocking density. Plant yield and nutrient profiles of fish showed no significant differences among treatments. No heavy metal accumulation was detected in fish tissues, confirming the environmental safety and sustainability of the integrated system.

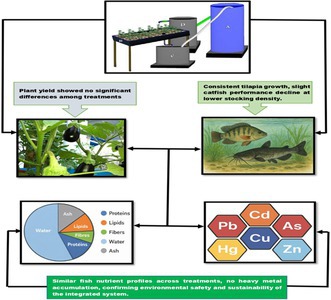

## 1 Introduction

Global production of aquatic animal food resources was estimated at 178 million tonnes in 2022, with contributions from capture fisheries (51%) and aquaculture (49%) (FAO 2024). In comparison, the aquaculture sector accounted for only 28% in 2000, against 72% from fisheries (FAO 2020). It was not until 2009 that aquaculture production equalled that of capture fisheries (Naylor et al. 2009). This rapid growth over the past two decades is largely attributed to the development and refinement of aquaculture, coupled with the stagnation of wild fish catches since the 1990s (FAO 2024). According to Lazard (2017), aquaculture is expected to remain the dominant source of fish for human consumption in the years ahead.

However, conventional aquaculture practices are increasingly criticized for their environmental impacts, particularly due to the discharge of nutrient‐rich effluents into surrounding ecosystems (Gowen et al. 1990). To address the challenge of sustainably increasing aquaculture production while mitigating environmental degradation, innovative and efficient production systems are being explored. One such system is aquaponics, which has emerged as a promising integrated farming technique with potential to serve as a foundation for future food production systems (Salam et al. 2014). Aquaponics is promoted as a sustainable alternative to conventional agriculture and aquaculture, with potential to enhance global and African food security (Obirikorang et al. 2021).

Aquaponics integrates recirculating aquaculture systems (RAS) and hydroponics, relying on a symbiotic relationship between fish, plants, and microorganisms (Somerville et al. 2014). Fish excrete waste, which is broken down by nitrifying bacteria into bioavailable nutrients for plants. In turn, plants absorb these nutrients and help purify the water, which is then recirculated back to the fish tanks (Rakocy 2012; Somerville et al. 2014).

In the Democratic Republic of Congo (DRC), previous studies by Kifufu (2019), Mafwila et al. (2019), and Lusasi et al. (2022) have identified Nile tilapia ( *Oreochromis niloticus* ) and African Catfish ( *Clarias gariepinus* ) as the most widely farmed species in conventional pond aquaculture systems. In light of efforts to introduce aquaponic practices in the DRC, the present study aims to assess the feasibility of co‐culturing these two species in an aquaponic system. Specifically, it evaluates whether such integration can be achieved without negatively impacting water quality, fish growth performance, nutritional composition, or posing risks of heavy metal contamination in fish tissues.

## 2 Material and Methods

### 2.1 Experimental Design and Culture Conditions

This trial was conducted over a period of 106 days, from July 8 to October 22, 2024, in the city of Bandundu, Democratic Republic of Congo. Ten identical aquaponic units were used, each stocked with a total initial biomass of 10 kg·m^−3^. The experimental species were Nile tilapia ( *Oreochromis niloticus* , 20.48 ± 0.29 g) and African Catfish ( *Clarias gariepinus* , 19.84 ± 0.46 g), both sourced from local hatcheries.

Five treatment groups were established, each reflecting a different stocking ratio between the two fish species, while maintaining a constant total biomass per tank:

- TC_1_: 2 kg·m^−3^ *O. niloticus* and 8 kg·m^−3^ *C. gariepinus*

- TC_2_: 4 and 6 kg·m^−3^ *O. niloticus* and *C. gariepinus*

- TC_3_: 5 and 5 kg·m^−3^ *O. niloticus* and *C. gariepinus*

- TC_4_: 6 kg·m^−3^ *O. niloticus* and 4 kg·m^−3^ *C. gariepinus*

- TC_5_: 8 kg·m^−3^ *O. niloticus* and 2 kg·m^−3^ *C. gariepinus*

Each tank was stocked with 100 fish in total. Fish were fed twice daily, at 09:00 and 15:00, using a commercial floating pellet containing 35% crude protein, 11.5% crude fat, 4% crude fiber, 8% ash, 0.2% sodium, 1.5% calcium, and 0.8% phosphorus.

Eggplant ( *Solanum melongena* ) was cultivated in the hydroponic section of the aquaponic system. Seedlings were raised in plastic pots and transplanted into the grow beds 30 days post‐germination, at a density of 9 plants/m^2^. All treatments followed the same planting and management procedures.

A detailed overview of the treatment arrangements is presented in Table 1.

**TABLE 1:** Fish and plant density in each experiment unit.

Treatments	TC_1_	TC_2_	TC_3_	TC_4_	TC_5_
Initial density (kg m^−3^)	10	10	10	10	10
Fish number/unit	100	100	100	100	100
Nile tilapia (no/unit)	20	40	50	60	80
African catfish (no/unit)	80	60	50	40	20
Initial biomass (g)	2021.8	2023.1	2029.7	2014.2	2021.5
Initial average weight (g)^a^	20.21	20.23	20.29	20.14	20.21
Initial biomass of Nile tilapia (g)	418.6	803.9	1020.4	1236.1	1635.7
Initial biomass of African catfish (g)	1603.2	1219.2	1009.3	778.1	385.8
Plant number per m^2^	9	9	9	9	9

^a^ Avarage of both species.

### 2.2 Data Collection

Ambient air temperature was recorded daily using a mercury thermometer. Water temperature, dissolved oxygen (DO), and pH were measured once per day using a multiparameter meter (Hanna HI98194). Total ammonia nitrogen (TAN) and nitrite (NO_2_^−^) concentrations were monitored twice per week using commercial colorimetric test kits.

For the plant component, data collection for *Solanum melongena* was limited to fruit parameters, including number of fruits, length (cm), diameter (cm), individual fruit weight (g), and total biomass (g). Harvesting began 56 days after transplanting and continued until the end of the trial. Fruits were harvested as they reached maturity.

Fish biometric data were collected at the beginning of the experiment (Day 0) and every 26 days thereafter, until the end of the 106‐day rearing period. At each sampling point, five fish of each species ( *O. niloticus* and *C. gariepinus* ) were randomly selected from each replicate. Measurements included standard length (mm) using an ichthyometer (precision: 0.1 mm) and body weight (g) using a digital scale (precision: 0.1 g).

The following growth performance indicators were calculated for each species:

- Weight Gain (WG) = Final weight − Initial weight
- Daily Weight Gain (DWG) = WG/Rearing period (days)
- Length Gain (LG) = Final length − Initial length
- Specific Growth Rate (SGR) = (lnFinalweight − lnInitialweight)/Rearing period (ln Final weight − ln Initial weight)/Rearing period (lnFinalweight − lnInitialweight)/Rearing period × 100
- Condition Factor (*K*) = (Body weight/Length (Andriani et al. 2017)) × 100

### 2.3 Biochemical Analysis

A proximate nutrient analysis was performed on both fish species at the end of the experiment. Five specimens of each species were randomly selected after harvest and sent to the Food Analysis Laboratory of the University of Kinshasa (UNIKIN) and the Congolese Control Office (OCC) laboratory for assessment of biochemical composition and heavy metal content.

The parameters analyzed included crude protein, crude fat, ash, moisture content, and concentrations of heavy metals in fish flesh. Both laboratories followed standard analytical procedures outlined by the Association of Official Analytical Chemists (AOAC 2020).

### 2.4 Statistical Analysis

All data are presented as means ± standard deviation (SD). Prior to analysis, Levene's test was used to verify the homogeneity of variances. One‐way analysis of variance (ANOVA) was applied to detect significant differences among treatment means, followed by Tukey's HSD test for post hoc pairwise comparisons.

Principal Component Analysis (PCA) was performed to assess the relationships between water quality variables and fish growth performance. Survival rates were compared using the Chi‐squared (*χ* ^2^) test. In addition, fish growth and production data were analyzed using generalized linear mixed‐effects models (GLMM) following the approach described by Sabwa et al. (2021).

All statistical analyses were performed using R software (version 4.3.2) and Excel equipped with the XLSTAT add‐in. A significance threshold of *p* < 0.05 was applied in all tests.

## 3 Results

### 3.1 Effects of Co‐Culture on Eggplant ( *Solanum melongena* ) Production

**FIGURE 1:**
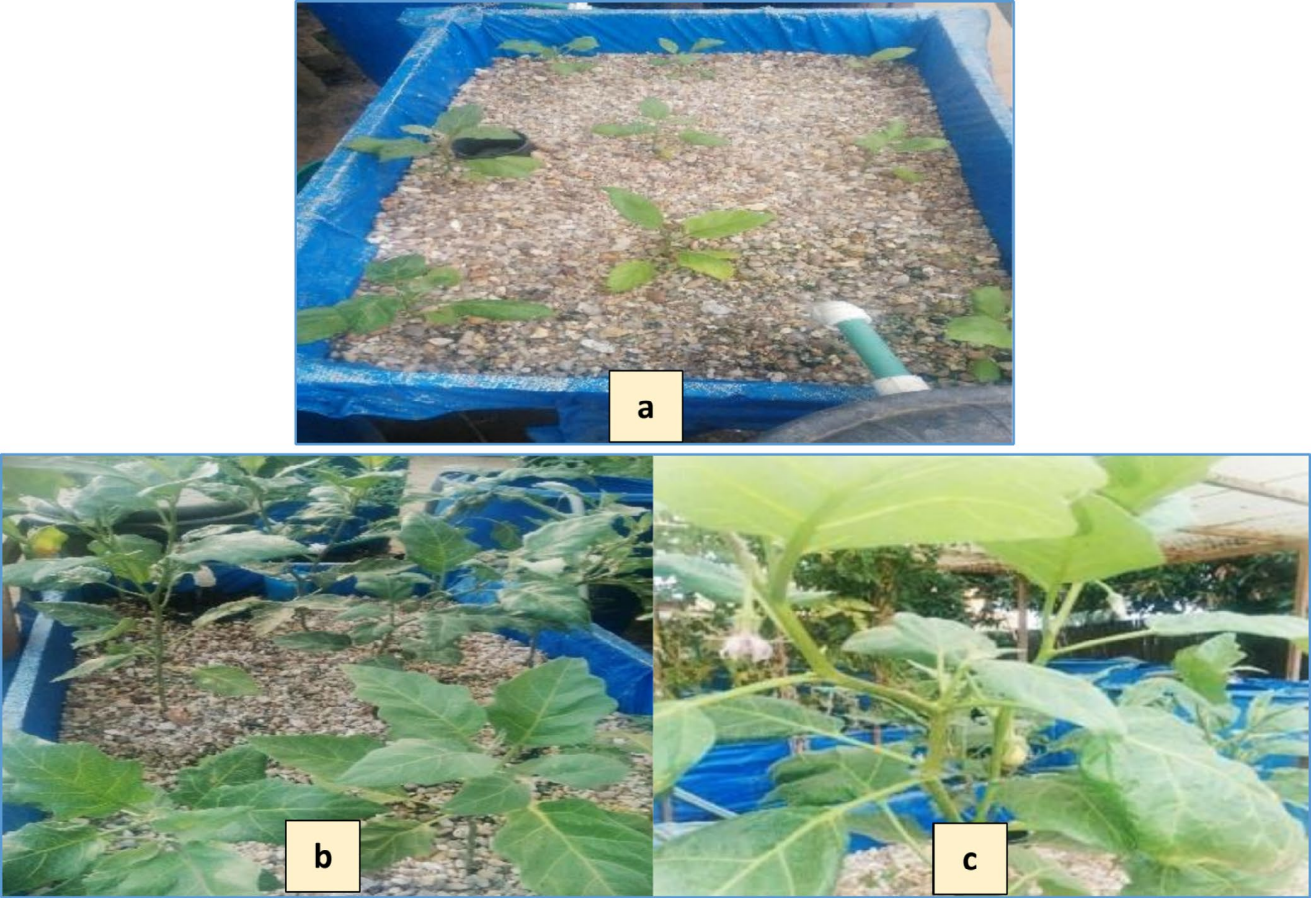
Eggplant developing roots after transplantation (a), in full vegetative growth (b), flowering and fruiting (c).

The vegetative development of *Solanum melongena* from transplanting to the onset of fruiting in the aquaponic systems is illustrated in Figure 1. Production parameters including number of fruits harvested, individual fruit weight, total fruit biomass, and marketable yield as influenced by the co‐culture of Nile tilapia and African catfish are summarized in Table 2.

**TABLE 2:** Production and yield performance of *S. melongena* in aquaponics.

Variant	Value in period (Week)							Marketable eggplant yield	
	1	2	3	4	5	6	7		
**Total number of fruits harvested**								**Nb/plant**	**Nb/m** ^**2**^ **/year**
TC_1_	16 ± 1.41^b^	22 ± 1.29	25 ± 1.89	18 ± 2.94^a^	23 ± 1.5	21 ± 1.70	18 ± 1.73^a^	7.9 ± 1.23	492.40 ± 8.5
TC_2_	19 ± 2.70^a^	20 ± 3.74	23 ± 1.25	13 ± 3.2^b^	22 ± 1.73	18 ± 2.38	16 ± 2.16^ab^	7.2 ± 1.52	451.08 ± 6.4
TC_3_	17 ± 1.27^ab^	23 ± 2.21	24 ± 82	22 ± 1.19^a^	20 ± 1.63	16 ± 1.43	13 ± 1.50^b^	7.5 ± 1.45	464.85 ± 5.8
TC_4_	18 ± 2.38^a^	23 ± 1.73	25 ± 1.50	14 ± 2.80^b^	17 ± 2.21	22 ± 1.73	23 ± 2.21^a^	7.8 ± 1.3	488.96 ± 7.3
TC_5_	22 ± 1.29^a^	24 ± 1.65	20 ± 1.63	17 ± 1.70^a^	19 ± 1.29	19 ± 1.25	18 ± 1.74^a^	7.7 ± 3.6	478.63 ± 6.9
p‐value	0.028	0.260	0.118	0.014	0.305	0.162	0.002	0.857	0.241
**Average fruit weight (g)**								**g/plant/week**	**g/m** ^**2**^ **/year**
TC_1_	64.5 ± 8.67^a^	51.8 ± 9.53^ab^	50.2 ± 4.82^a^	52.9 ± 7.49	44.8 ± 5.21^bc^	57.2 ± 7.40	53.1 ± 4.84^a^	53.5 ± 6.4	1289.55 ± 73.29
TC_2_	66.7 ± 9.13^a^	50.3 ± 6.86^ab^	55.0 ± 7.15^a^	59.3 ± 7.83	43.5 ± 5.92^c^	53.7 ± 7.39	47.9 ± 5.16^ab^	54.2 ± 8.3	1305.42 ± 57.84
TC_3_	69.2 ± 7.65^a^	57.4 ± 8.47^a^	46.2 ± 6.52^ab^	54.5 ± 4.74	57.8 ± 7.38^a^	62.4 ± 6.17	53.2 ± 8.43^a^	57.2 ± 7.8	1379.76 ± 85.61
TC_4_	65.3 ± 8.38^a^	52.7 ± 7.34^ab^	43.2 ± 4.98^b^	60.8 ± 9.53	53.9 ± 6.73^ab^	47.2 ± 6.49	40.8 ± 4.97^b^	51.9 ± 5.7	1253.05 ± 64.10
TC_5_	47.3 ± 5.92^b^	46.2 ± 7.43^b^	49.8 ± 4.71^a^	62.1 ± 6.84	63.2 ± 8.24^a^	60.1 ± 8.49	57.1 ± 6.36^a^	55.1 ± 6.3	1328.46 ± 79.65
p‐value	0.019	0.047	0.005	0.834	0.001	0.583	0.001	0.280	0.094
**Total Biomass (g)**								**g/m** ^**2**^ **/week**	**g/m** ^**2**^ **/year**
TC_1_	1032.8 ± 65.39^b^	1139.6 ± 54.8^b^	1255.4 ± 70.5^a^	952.2 ± 39.6^b^	1030.4 ± 43.6^b^	991.2 ± 36.4^b^	955.8 ± 27.9^b^	1051.0 ± 51.3	22334.4 ± 78.4
TC_2_	1267.5 ± 72.90^a^	1006.2 ± 49.2^c^	1205.6 ± 83.1^a^	770.9 ± 23.2^c^	957.7 ± 35.0^c^	966.9 ± 40.8^b^	766.4 ± 23.5^c^	1163.8 ± 57.9	28052.6 ± 65.9
TC_3_	1176.7 ± 61.05^a^	1327.9 ± 73.5^a^	1108.8 ± 69.6^b^	1199.4 ± 55.7^a^	1156.2 ± 52.8^a^	998.4 ± 43.5^b^	691.6 ± 25.8^c^	1094.1 ± 43.2	26372.9 ± 82.7
TC_4_	1175.4 ± 82.61^a^	1212.8 ± 68.3^a^	1080.5 ± 74.2^b^	851.2 ± 31.4^bc^	916.3 ± 46.3^c^	1034.4 ± 56.^3ab^	938.4 ± 32.4^b^	1029.8 ± 48.5	24823.4 ± 85.2
TC_5_	1040.6 ± 60.73^b^	1108.8 ± 62.7^b^	996.3 ± 77.9^c^	1055.7 ± 46.2^b^	1200.8 ± 42.8^a^	1141.9 ± 48.5^a^	1027.8 ± 39.7^a^	1081.7 ± 41.9	26073.0 ± 83.6
*p*	0.027	0.008	0.013	0.007	0.009	0.004	0.000	0.081	0.431

*Note:* Values with the different superscript letters are significantly different *p* < 0.05. Yield = Biomass × 365/Experiment duration × cultures surface.

The total number of fruits harvested per week varied across treatments during the seven‐week harvesting period, ranging from 13 ± 3.1 fruits in TC_2_ (Week 4) to 25 ± 1.29 fruits in TC_1_ (Week 3) (Table 2). The mean number of fruits per plant ranged between 7.0 ± 1.73 and 7.5 ± 1.45. One‐way ANOVA revealed no significant differences in the number of fruits harvested per plant among treatments (*p* > 0.05).

Regarding average fruit weight per plant, values ranged from 54.0 ± 8.30 to 63.60 ± 6.40 g. Table 2 shows that the lowest mean fruit weight was observed in TC_4_ during Week 3 (43.2 ± 4.98 g), while the highest was recorded in TC_3_ during Week 1 (69.2 ± 7.65 g). However, no statistically significant differences were found between treatments (*p* > 0.05).

The marketable fruit yield, expressed as annual biomass production per square meter, was as follows: TC_1_: 22334.4 ± 78.4; TC_2_: 28052.6 ± 65.9; TC_3_: 26372.9 ± 82.7; TC_4_: 24823.4 ± and TC_5_: 26073.0 ± 83.6 g/m^2^/year. Analysis of variance showed no significant difference in marketable fruit biomass among treatments (*p* > 0.05), consistent with the absence of significant differences in number of fruits and mean fruit weight (*p* > 0.05).

**FIGURE 2:**
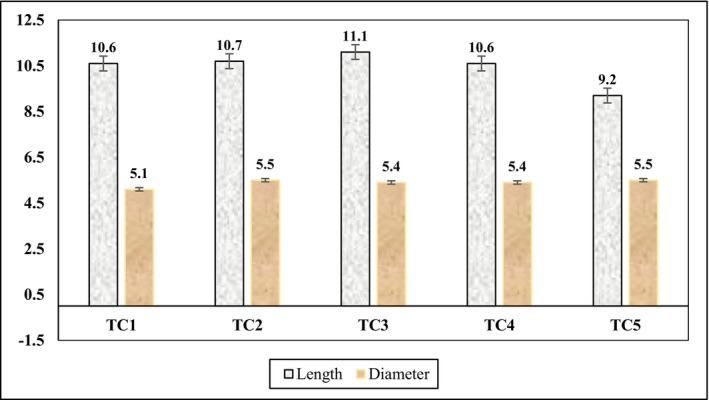
Average length and diameters of eggplant fruit in cm.

The mean fruit length varied slightly among treatments, with values of 10.6 cm (TC_1_), 10.7 cm (TC_2_), 11.1 cm (TC_3_), 10.6 cm (TC_4_), and 9.2 cm (TC_5_). Similarly, mean fruit diameter ranged from 5.2 to 5.5 cm across treatments (Figure 2). ANOVA revealed no significant differences in either fruit length or diameter (*p* > 0.05).

### 3.2 Growth Performance and Survival Rate of Fish

#### 3.2.1 Effect of Co‐Culture on Fish Survival Rate

The survival rates of fish throughout the experimental period are presented in Table 3. Chi‐squared analysis revealed no statistically significant differences in survival rates among treatments (*p* > 0.05), indicating that co‐culture ratios had no measurable impact on fish survival at the population level. However, a paradoxical trend was observed: the lowest survival rate of Nile Tilapia was recorded in TC_1_, where the species was stocked at its lowest proportion, while the lowest survival of African catfish was noted in TC_5_, where catfish were least represented. This suggests that stocking each species in very low proportions may affect their individual survival, despite no statistical difference across overall treatments (*p* > 0.05).

**TABLE 3:** Survival rate of fish during the experiment period.

Fish species	Experiment treatments					*p*
	TC_1_	TC_2_	TC_3_	TC_4_	TC_5_	
Nile tilapia	92.5 ± 5.7	95.0 ± 6.3	95.0 ± 6.9	96.7 ± 5.5	96.8 ± 4.2	0.093
African catfish	98.7 ± 2.9	95.0 ± 3.8	97.0 ± 4.5	97.5 ± 3.1	90.0 ± 4.5	0.158

#### 3.2.2 Effects of Co‐Culture on Fish Growth Parameters

**FIGURE 3:**
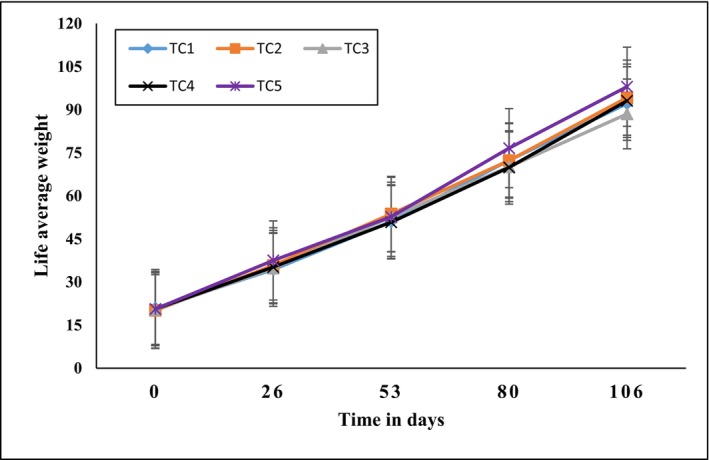
Changes in the average live body weight of Nile tilapia.

**FIGURE 4:**
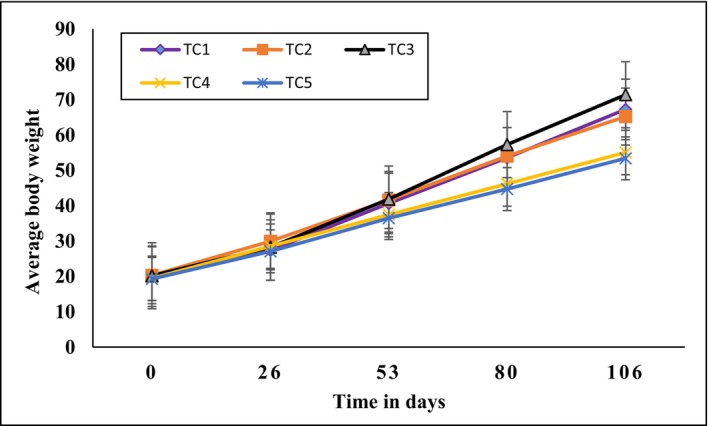
Changes in the average live body weight of African catfish.

Fish growth data for both species over the entire experimental period are presented in Table 4. The progression of average live weight for Nile tilapia and African catfish is illustrated in Figures 3 and 4, respectively. One‐way ANOVA results indicated that co‐culture treatments had no significant effect on the growth of Nile Tilapia (*p* > 0.05). In contrast, some growth parameters of African catfish showed significant differences among treatments.

**TABLE 4:** Growth performance of Nile tilapia and African catfish cultured in association in aquaponics.

Variables	Nile Tilapia						African catfish					
	TC_1_	TC_2_	TC_3_	TC_4_	TC_5_	*p*	TC_1_	TC_2_	TC_3_	TC_4_	TC_5_	*p*
**Period**	**Weight gain (g)**											
0–26 days	13.4 ± 3.2	15.7 ± 2.7	14.4 ± 3.3	14.6 ± 2.5	16.9 ± 3.8	0.073	7.4 ± 1.30	9.6 ± 0.90	8.1 ± 1.74	9.1 ± 1.37	7.8 ± 1.65	0.539
27–53 days	16.7 ± 4.6	17.9 ± 3.2	17.8 ± 5.7	15.6 ± 4.0	15.2 ± 4.1	0.197	13.2 ± 3.8	11.7 ± 2.2	13.6 ± 3.8	8.9 ± 2.42	9.4 ± 2.97	0.281
54–80 days	21.3 ± 3.7	18.6 ± 2.9	17.5 ± 4.3	19.1 ± 3.9	23.9 ± 5.5	0.063	12.9 ± 3.2^a^	12.4 ± 3.9^a^	15.4 ± 4.7^a^	8.7 ± 1.85^b^	8.2 ± 2.45^b^	0.002
81–106 days	19.8 ± 4.6	21.9 ± 5.4	18.4 ± 5.9	23.2 ± 5.2	21.4 ± 4.3	0.640	13.7 ± 4.0^a^	11.2 ± 4.1^a^	14.1 ± 3.2^a^	8.9 ± 1.05^b^	8.7 ± 3.83^b^	0.205
0–106 days	71.2 ± 5.2	74.1 ± 3.3	68.5 ± 4.7	72.5 ± 6.7	77.4 ± 5.8	0.279	47.8 ± 6.8^a^	44.9 ± 7.2^a^	51.2 ± 8.6^a^	35.6 ± 6.5^b^	34.1 ± 7.2^b^	0.001
**Period**	**Daily weight gain (g/day)**											
0–26 days	0.51 ± 0.01	0.60 ± 0.01	0.44 ± 0.00	0.56 ± 0.01	0.65 ± 0.03	0.584	0.28 ± 0.01	0.28 ± 0.00	0.36 ± 0.02	0.35 ± 0.02	0.30 ± 0.01	0.842
27–53 days	0.64 ± 0.02	0.70 ± 0.03	0.66 ± 0.02	0.79 ± 0.02	0.58 ± 0.01	0.087	0.50 ± 0.03^a^	0.51 ± 0.04^a^	0.45 ± 0.03^a^	0.34 ± 0.03^b^	0.36 ± 0.02^b^	0.020
54–80 days	0.81 ± 0.03	0.71 ± 0.02	0.67 ± 0.02	0.73 ± 0.01	0.92 ± 0.04	0.213	0.49 ± 0.02^a^	0.48 ± 0.02^b^	0.47 ± 0.01^b^	0.33 ± 0.00^c^	0.31 ± 0.00^c^	0.001
81–106 days	0.76 ± 0.02	0.69 ± 0.02	0.86 ± 0.03	0.89 ± 0.03	0.82 ± 0.02	0.438	0.52 ± 0.03^a^	0.52 ± 0.02^a^	0.43 ± 0.02^b^	0.34 ± 0.01^bc^	0.33 ± 0.01^c^	0.010
0–106 days	0.67 ± 0.01	0.64 ± 0.02	0.62 ± 0.03	0.68 ± 0.02	0.73 ± 0.03	0.752	0.45 ± 0.07^a^	0.44 ± 0.03^a^	0.42 ± 0.02^a^	0.33 ± 0.02^b^	0.32 ± 0.04^a^	0.031
**Period**	**Length gain (cm)**											
0–26 days	1.75 ± 0.83	2.12 ± 1.02	1.53 ± 0.12	1.90 ± 0.64	1.85 ± 0.87	0.064	1.97 ± 0.27	2.04 ± 1.04	1.52 ± 0.14	1.12 ± 0.18	1.20 ± 0.83	0.628
27–53 days	2.19 ± 1.27^a^	2.96 ± 1.28^a^	1.09 ± 0.24^b^	1.64 ± 1.19^a^	1.93 ± 1.35^a^	0.031	1.84 ± 0.41^a^	1.48 ± 0.03^b^	1.57 ± 1.07^b^	1.07 ± 0.13^c^	1.12 ± 0.69^c^	0.012
54–80 days	1.97 ± 0.61	1.85 ± 0.93	1.97 ± 0.17	2.47 ± 1.06	2.86 ± 1.10	0.438	1.95 ± 0.23^a^	1.62 ± 0.06^b^	1.45 ± 0.09^c^	1.24 ± 0.82^d^	1.17 ± 0.31^d^	0.001
81–106 days	2.54 ± 1.08	2.90 ± 0.87	1.89 ± 0.18	2.86 ± 1.81	1.74 ± 1.63	0.763	2.45 ± 1.09^a^	2.41 ± 0.94^a^	1.99 ± 1.62^b^	1.15 ± 0.71^c^	1.19 ± 0.72^c^	0.003
0–106 days	8.45 ± 2.62	9.83 ± 2.49	6.48 ± 2.83	8.93 ± 3.65	8.38 ± 3.5	0.096	8.21 ± 1.35^a^	7.55 ± 1.79^a^	6.53 ± 2.02^b^	4.58 ± 1.24^c^	4.68 ± 1.85^c^	0.001
**Period**	**Specific growth rate (%/day)**											
0–26 days	0.48 ± 0.02^a^	0.51 ± 0.01^a^	0.29 ± 0.00^b^	0.49 ± 0.03^a^	0.53 ± 0.03^a^	0.001	0.25 ± 0.00	0.29 ± 0.03	0.31 ± 0.02	0.31 ± 0.01	0.23 ± 0.00	0.219
27–53 days	0.53 ± 0.02	0.65 ± 0.03	0.42 ± 0.01	0.58 ± 0.01	0.45 ± 0.02	0.760	0.27 ± 0.00^b^	0.25 ± 0.03^b^	0.37 ± 0.02^a^	0.29 ± 0.00^b^	0.21 ± 0.00^b^	0.040
54–80 days	0.67 ± 0.07^a^	0.63 ± 0.07^b^	0.49 ± 0.02^b^	0.53 ± 0.05^b^	0.74 ± 0.04^a^	0.042	0.28 ± 0.01	0.23 ± 0.00	0.31 ± 0.01	0.38 ± 0.03	0.25 ± 0.01	0.527
81–106 days	0.58 ± 0.03	0.60 ± 0.5	0.56 ± 0.06	0.61 ± 0.04	0.67 ± 0.05	0.543	0.29 ± 0.00	0.21 ± 0.00	0.38 ± 0.03	0.31 ± 0.01	0.29 ± 0.01	0.139
0–106 days	0.59 ± 0.05	0.61 ± 0.4	0.57 ± 0.03	0.56 ± 0.04	0.52 ± 0.02	0.032	0.27 ± 0.01	0.24 ± 0.01	0.33 ± 0.02	0.31 ± 0.02	0.24 ± 0.01	0.306
**Period**	**Factor *K***											
0–26 days	1.5 ± 0.36	1.8 ± 0.17	1.6 ± 0.23	1.9 ± 0.10	1.6 ± 0.34	0.354	1.9 ± 0.26	1.7 ± 0.25	1.8 ± 0.14	1.2 ± 0.26	1.3 ± 0.25	0.369
27–53 days	2.3 ± 1.03	1.4 ± 0.23	2.4 ± 0.68	2.3 ± 0.74	2.3 ± 1.27	0.078	1.7 ± 0.33	1.4 ± 0.13	1.5 ± 0.37	1.6 ± 0.10	1.7 ± 0.43	0.425
54–80 days	1.7 ± 0.21^b^	2.7 ± 0.28^a^	2.6 ± 1.13^a^	2.1 ± 1.11^a^	2.0 ± 0.71^b^	0.004	2.2 ± 1.28^a^	1.7 ± 0.17^a^	1.3 ± 0.12^ab^	1.4 ± 0.14^ab^	1.2 ± 0.12^b^	0.041
81–106 days	2.5 ± 0.86	2.3 ± 0.32	2.3 ± 1.27	2.6 ± 1.37	2.1 ± 1.08	0.670	1.6 ± 0.21	1.2 ± 0.34	1.8 ± 0.28	1.7 ± 0.29	1.3 ± 0.16	0.210
0–106 days	2.1 ± 0.48	1.9 ± 0.37	1.8 ± 0.42	2.3 ± 1.03	1.9 ± 0.72	0.653	1.3 ± 0.19	1.5 ± 0.28	1.3 ± 0.12	1.4 ± 0.14	1.3 ± 0.19	0.322

*Note:* The values of on row with the different superscript are significantly different (*p* < 0.05).

For Nile tilapia, mean live weight increased from 20.93 ± 5.8 to 92.13 ± 16.3 g in TC_1_, 20.09 ± 4.5 to 94.19 ± 13.9 g in TC_2_, 20.41 ± 5.1 to 88.51 ± 15.4 g in TC_3_, 20.6 ± 3.9 to 93.16 ± 18.6 g in TC_4_ and 20.6 ± 6.1 to 98.0 ± 12.5 g in TC_5_. Figure 3 depicts the evolution of Nile tilapia mean weight over time. Statistical analysis showed no significant differences in mean live weight among treatments for Nile tilapia (*p* > 0.05).

For African catfish, the mean body weights increased from 20.04 ± 4.1 to 67.24 ± 11.7 g in TC_1_, from 20.32 ± 5.2 to 65.22 ± 12.5 g in TC_2_, from 20.18 ± 4.9 to 71.38 ± 13.6 g in TC_3_, from 19.45 ± 5.1 to 55.05 ± 10.8 g in TC_4_, and from 19.29 ± 4.8 to 53.39 ± 13.9 g in TC_5_.

Statistical analysis indicated that fish in TC_3_ achieved significantly higher weights compared to those in TC_4_ and TC_5_. No significant differences were found between TC_1_, TC_2_, and TC_3_. The divergence in body weight between TC_3_ and TC_4_ became apparent from Day 26 and continued throughout the experiment, culminating in a pronounced difference by the trial's end. The progression of average body weight over time is illustrated in Figure 4.

Table 4 presents the mean weight gain of Nile Tilapia during the experiment. From day 0 to day 26, it ranged from 13.4 ± 3.2 to 16.9 ± 3.8 g (TC_5_). Between days 27 and 53, the fish recorded gains from 15.2 ± 4.1 g (TC_5_) to 17.9 ± 3.2 g (TC_2_). From days 54 to 80, the gains varied between 17.5 ± 4.3 g (TC_3_) and 23.9 ± 5.5 g (TC_5_). In the final phase (days 81–106), values ranged from 19.8 ± 4.6 g (TC_1_) to 23.2 ± 5.2 g (TC_4_). Over the entire experimental period (0–106 days), cumulative weight gains were 71.2 ± 5.2 g (TC_1_), 68.1 ± 3.3 g (TC_2_), 66.4 ± 4.7 g (TC_3_), 72.5 ± 6.7 g (TC_4_), and 77.4 ± 5.0 g (TC_5_). Analysis of variance revealed no significant differences among treatments, either within the different growth periods or for the overall duration of the experiment.

Daily weight gain ranged from 0.44 ± 0.00 g/day (TC_3_, 0–26 days) to 0.92 ± 0.04 g/day (TC_5_, 5, respectively, 4–80 days). For the full experimental period (0–106 days), mean daily weight gains were 0.67 ± 0.01 g/day (TC_1_), 0.64 ± 0.02 g/day (TC_2_), 0.62 ± 0.03 g/day (TC_3_), 0.68 ± 0.02 g/day (TC_4_), and 0.73 ± 0.03 g/day (TC_5_), with no significant differences. Mean length gain varied between 1.75 ± 0.83 g/day (TC_1_, 0–26 days) and 2.96 ± 1.28 g/day (TC_2_, 27–53 days). Significant differences were observed only during this second period. Overall, from day 0 to day 106, mean length gains were 8.45 ± 2.62 g/day (TC_1_), 9.83 ± 2.49 g/day (TC_2_), 6.48 ± 2.83 g/day (TC_3_), 8.93 ± 3.65 g/day (TC_4_), and 8.38 ± 3.50 g/day (TC_5_).

The specific growth rate (SGR) ranged from 0.29%/day ± 0.00%/day (TC_3_, 0–26 days) to 0.74%/day ± 0.04%/day (TC_5_, 54–80 days). Mean values over the entire period were 0.59%/day ± 0.05%/day (TC_1_), 0.61%/day ± 0.04%/day (TC_2_), 0.57%/day ± 0.03%/day (TC_3_), 0.56%/day ± 0.04%/day (TC_4_), and 0.52%/day ± 0.02%/day (TC_5_). The highest condition factor (*K*) was recorded with TC_1_ (2.7 ± 0.28) and TC_2_ (2.6 ± 1.13). Overall, *K* values averaged 2.1 ± 0.48 (TC_1_), 1.9 ± 0.37 (TC_2_), 1.8 ± 0.42 (TC_3_), 2.3 ± 1.03 (TC_4_), and 1.9 ± 0.72 (TC_5_). No significant differences were detected among treatments.

Table 4 shows that the growth performance of African catfish tended to decline as stocking density in the aquaponic tanks decreased. From day 27 onward, treatments with higher catfish numbers (TC_1_, TC_2_, and TC_3_) exhibited greater weight gains (13.2 ± 3.8, 11.7 ± 2.2, and 13.6 ± 3.8 g, respectively) compared with TC_4_ (8.9 ± 2.42 g) and TC_5_ (9.4 ± 2.97 g). This trend persisted until the end of the experiment, with mean weight gains over 106 days of 47.8 ± 6.8, 44.9 ± 7.2, 51.2 ± 8.6, 35.6 ± 6.5, and 34.1 ± 7.2 g for TC_1_, TC_2_, TC_3_, TC_4_, and TC_5_, respectively.

Co‐culture also had a modest effect on daily weight gain. During the first 26 days, daily growth ranged from 0.28 ± 0.00 g/day (TC_2_) to 0.36 ± 0.02 g/day (TC_3_). After day 27, fish in TC_1_ (0.45 ± 0.07 g/day), TC_2_ (0.44 ± 0.03 g/day), and TC_3_ (0.42 ± 0.02 g/day) consistently outperformed those in TC_4_ (0.33 ± 0.02 g/day) and TC_5_ (0.32 ± 0.04 g/day). A similar pattern was observed for length gain, with higher increases in TC_1_–TC_3_ (8.21 ± 1.35, 7.55 ± 1.79, and 6.53 ± 2.02 cm, respectively) than in TC_4_ (4.58 ± 1.24 cm) and TC_5_ (4.68 ± 1.85 cm).

Statistical analysis confirmed significant differences among treatments. The mean specific growth rate across the experimental period was 0.27% ± 0.01%/day for TC_1_, 0.24% ± 0.01%/day for TC_2_, 0.33% ± 0.02%/day for TC_3_, 0.31% ± 0.02%/day for TC_4_, and 0.24% ± 0.01%/day for TC_5_. By contrast, condition factor (*K*) values did not differ significantly (*p* > 0.05) among treatments, ranging from 1.3 ± 0.12 to 1.5 ± 0.28.

To further assess the impact of co‐culture on growth performance, generalized linear mixed‐effects models (GLMM) were applied for both species. Results are summarized in Tables 5 and 6. GLMM analysis confirmed that co‐culture did not significantly affect growth variables of Nile Tilapia (Table 5). Conversely, significant effects were detected for African catfish growth parameters (Table 6).

**TABLE 5:** Results of generalized mixed effects models for Nile tilapia over the experimental period (106 days).

	WG (g)	WL (cm)	DGR (g/day)	SGR (%/day)	*K*
**Fixed effects**	***β* (SE), *t*‐value**	***β* (SE), *t*‐value**	***β* (SE), *t*‐value**	***β* (SE), *t*‐value**	***β* (SE), *t*‐value**
Intercept	8.26 (0.17), 14.79	2.53 (0.12), 15.8**	1.37 (0.04), 84.9	1.34 (0.21), 74.9	1.97 (0.02), 54.3*
Fish coculture	1.09 (0.05), 2.74	0.18 (0.01), 1.03	0.69 (0.11), 27.3	0.67 (0.03), −2.4	0.39 (0.21), 45.4
Time in days	6.84 (0.003), 21.07**	0.04 (0.005), −0.61**	0.08 (0.001), −4.9***	0.09 (0.004), 9.4	0.15 (0.03), 24.1***
Fish coculture × Time	0.137 (0.002), 15.06**	0.003 (0.002), 1.86***	0.102 (0.13), 3.8**	1.19 (0.04), −14.9	0.01 (0.001), 3.3**
**Anova for fixed effects**	***F*‐value**	***F*‐value**	***F*‐value**	***F*‐value**	***F*‐value**
Intercept	52141.9	4712.6	1268.0	1904.1	1821.4
Fish coculture	2181.4	2439.1	498.4	814.9	547.2
Time in days	1812.84**	1524.8***	307.1*	207.4**	109.4***
Fish coculture × Time	167.1	1249.6	79.4	3.9	9.3
**Random effect**					
Tank (intercept) SD	0.92	0.17	< 0.001	0.93	0.181
Residual SD	0.128	1.22	0.083	1.47	0.07
*R* ^2^ GLMM (m)	0.84	0.74	0.59	0.83	0.02
*R* ^2^ GLMM (c)	0.57	0.51	0.12	0.16	0.14

*Note:* The full model included all treatments tested, time in weeks, and interactive effects coculture × time as fixed effects and rearing tank as a random effect. The marginal *R* ^2^ (GLMM (m); fixed effects only) and the conditional *R* ^2^ (GLMM (c); fixed and random effects) represent the proportion variance explained by each model.

Abbreviations: SD, standard deviation; SE, standard error.

* *p* < 0.05.

** *p* < 0.01.

*** *p* < 0.001.

**TABLE 6:** Results of generalized mixed effects models for African catfish over the experimental period (106 days).

	Weight (g)	Length (cm)	DGR (g)	SGR (%/day)	*K*
**Fixed effects**	***β* (SE), *t*‐value**	***β* (SE), *t*‐value**	***β* (SE), *t*‐value**	***β* (SE), *t*‐value**	***β* (SE), *t*‐value**
Intercept	23.50 (2.14), 1.69**	5.02 (0.10), 12.4***	1.09 (0.12), 8.41**	3.20 (2.14), 1.31***	0.007 (0.03), 2.14**
Fish coculture	0.39 (0.32), 4.9**	0.11 (1.43), 3.9**	0.03 (0.01), 1.32***	0.18 (0.12), 0.05**	0.002 (0.001), 0.17**
Time in days	2.4 (0.2), 7.82**	−0.32 (12), 0.09***	0.05 (1.32), 1.55***	0.01 (0.001), 2.14*	0.14 (0.006), 2.24**
Fish coculture × Time	0.76 (2.19), 0.12***	0.42 (0.01), 1.57	0.86 (0.39), 0.35**	0.49 (0.32), 0.36*	0.08 (0.001), 0.06*
**Anova for fixed effects**	***F*‐value**	***F*‐value**	***F*‐value**	***F*‐value**	***F*‐value**
Intercept	143.9**	374.1***	57.4***	79.5**	26.1**
Fish coculture	0.52	147.4***	38.3*	0.17*	1.3***
Time in days	44.9**	7.10***	61.5**	1.69*	0.03***
Fish coculture × Time	3.1*	0.21**	14.3*	0.09*	0.19
**Random effects**					
Tank (intercept), SD	0.01	0.07	< 0.001	0.013	0.42
Residual SD	12.7	4.86	0.32	3.94	0.54
*R* ^2^ GLMM (m)	0.39	0.03	0.47	0.04	0.12
*R* ^2^ GLMM (c)	0.16	0.10	0.19	0.32	0.01

*Note:* The full model included all treatments tested, time in weeks and interactive effects coculture × time as fixed effects and rearing tank as a random effect. The marginal *R* ^2^ (GLMM (m); fixed effects only) and the conditional *R* ^2^ (GLMM (c); fixed and random effects) represent the proportion variance explained by each model.

Abbreviations: SD, standard deviation; SE, standard error.

* *p* < 0.05.

** *p* < 0.01.

*** *p* < 0.0.

For both species, GLMM indicated a positive effect of time and the interaction between time and co‐culture on fish growth, with random effects showing limited influence on the measured parameters. These findings demonstrate that fish growth improved over time regardless of treatment, with co‐culture ratios particularly influencing African catfish growth.

### 3.3 Biochemical Analysis Results

#### 3.3.1 Effects of Co‐Culture on Nutritional Body Composition of Fish

The proximate body composition of Nile tilapia and African catfish under different treatments is presented in Table 7. For Nile tilapia, moisture content ranged from 77.4% ± 1.1% (TC_4_) to 87.6% ± 1.3% (TC_5_). Crude protein levels varied between 24.0% ± 1.6% (TC_4_) and 25.0% ± 2.5% (TC_2_), while crude lipids ranged from 3.05% ± 0.1% (TC_3_) to 3.57% ± 0.3% (TC_1_). Ash content was between 0.81% ± 0.0% (TC_1_) and 0.91% ± 0.1% (TC_4_–TC_5_), and crude fiber between 1.06% ± 0.0% (TC_4_) and 1.42% ± 0.1% (TC_2_). Statistical analysis revealed no significant differences (*p* > 0.05) among treatments for all proximate composition parameters. For African catfish, moisture content ranged from 82.5% ± 1.4% (TC_4_) to 87.2% ± 2.6% (TC_1_). Crude protein values varied between 15.5% ± 1.4% (TC_4_) and 19.8% ± 1.6% (TC_3_). Crude lipids were relatively stable, ranging from 6.15% ± 0.7% (TC_4_) to 7.32% ± 1.0% (TC_3_). Ash content varied between 0.94% ± 0.0% (TC_5_) and 1.64% ± 0.2% (TC_1_), while crude fiber ranged from 1.56% ± 0.1% (TC_5_) to 1.90% ± 0.2% (TC_4_). As with tilapia, no significant differences (*p* > 0.05) were observed among treatments for any of the proximate composition variables.

**TABLE 7:** Nutritional body composition of fish.

Fish species	Treatments	Proximate body composition				
		Moisture (%)	Crude protein (%)	Crude lipids (%)	Ash (%)	Crude fiber (%)
Nile tilapia	TC_1_	86.9 ± 1.2	25.0 ± 1.2	3.57 ± 0.3	0.81 ± 0.0	1.39 ± 0.1
	TC_2_	79.8 ± 1.1	25.0 ± 2.5	3.29 ± 0.1	0.82 ± 0.0	1.42 ± 0.1
	TC_3_	85.7 ± 1.4	24.3 ± 1.8	3.05 ± 0.1	0.86 ± 0.0	1.27 ± 0.1
	TC_4_	77.4 ± 1.1	24.0 ± 1.6	3.57 ± 0.2	0.91 ± 0.1	1.06 ± 0.0
	TC_5_	87.6 ± 1.3	24.2 ± 2.3	3.19 ± 0.1	0.91 ± 0.1	1.34 ± 0.1
	*p*	0.092	0.320	0.085	0.713	0.531
African catfish	TC_1_	87.2 ± 2.6	18.6 ± 1.5	6.72 ± 0.2	1.64 ± 0.2	1.78 ± 0.3
	TC_2_	83.5 ± 1.8	17.2 ± 1.1	6.99 ± 0.6	0.97 ± 0.1	1.84 ± 0.1
	TC_3_	84.9 ± 2.6	19.8 ± 1.6	7.32 ± 1.0	1.05 ± 0.1	1.69 ± 0.1
	TC_4_	82.5 ± 1.4	15.5 ± 1.4	6.15 ± 0.7	1.18 ± 0.1	1.90 ± 0.2
	TC_5_	85.9 ± 1.7	17.9 ± 1.6	6.28 ± 0.5	0.94 ± 0.0	1.56 ± 0.1
	*p*	0.184	0.202	0.897	0.081	0.874

#### 3.3.2 Effects of Co‐Culture on Heavy Metal Concentrations in Fish Flesh

Concentrations of seven heavy metals in the flesh of Nile Tilapia and African Catfish are reported in Table 8. Lead (Pb), cadmium (Cd), and arsenic (As) were not detected in any sample for both species. Trace amounts of mercury (Hg), manganese (Mn), copper (Cu), and zinc (Zn) were detected. For Nile Tilapia, mean concentrations followed the order: Mn > Cu > Zn > Hg, with average values of: Mn: 0.086, Cu: 0.034, Zn: 0.025 and Hg: 0.014 mg/kg. Similar patterns were observed in African catfish, with concentrations comparable to those in Nile Tilapia. All detected metal concentrations were well below internationally accepted safety limits. ANOVA revealed no significant differences in heavy metal concentrations among treatments for either species (*p* > 0.05).

**TABLE 8:** Heavy metals concentration in flesh of fish (mg/kg).

Elements	Experimental treatments					Standard values^a^
	TC_1_	TC_2_	TC_3_	TC_4_	TC_5_	
**Nile tilapia**						
Lead (Pb)	0.00	0.00	0.00	0.00	0.00	0.20
Cadmium (Cd)	0.00	0.00	0.00	0.00	0.00	0.05
Mercury (Hg)	0.014	0.013	0.016	0.014	0.015	0.50
Arsenic (As)	0.00	0.00	0.00	0.00	0.00	0.10
Manganese (Mn)	0.091	0.084	0.079	0.087	0.093	0.10
Copper (Cu)	0.039	0.034	0.029	0.033	0.035	0.10
Zinc (Zn)	0.025	0.029	0.022	0.028	0.024	100
**African catfish**						
Lead (Pb)	0.00	0.00	0.00	0.00	0.00	0.20
Cadmium (Cd)	0.00	0.00	0.00	0.00	0.00	0.05
Mercury (Hg)	0.011	0.010	0.014	0.009	0.013	0.50
Arsenic (As)	0.00	0.00	0.00	0.00	0.00	0.10
Manganese (Mn)	0.059	0.07	0.062	0.067	0.063	0.10
Copper (Cu)	0.026	0.031	0.029	0.034	0.031	0.10
Zinc (Zn)	0.033	0.045	0.032	0.036	0.039	100

^a^ Based on FAO (2020) and WHO (2006).

### 3.4 Analysis of Water and Environmental Parameters

#### 3.4.1 Effects of Co‐Culture on Water Quality

The main water quality parameters measured during the trial are summarized in Table 9. Overall, the recorded values remained within ranges considered suitable for aquaponic production and for the growth of Nile tilapia and African catfish.

**TABLE 9:** Water and air quality during the trial.

	DO		pH		T° water		T° Air		EC		NH_3_/NH_4_^+^		NO_2_^−^		NO_3_^−^	
	HP	AP	HP	AP	HP	AP	HP	AP	HP	AP	HP	AP	HP	AP	HP	AP
**TC**_**1**_																
Mean	6.43	7.80	7.43	7.27	30.62	30.18	—	30.85	952.9	765.3	0.09	0.76	< 0.05	< 0.05	1.43	4.86
SD	1.17	2.57	1.07	1.12	5.76	7.21	—	4.52	34.6	21.0	0.03	0.04	0.00	0.00	0.07	1.28
*N*	106	106	106	106	106	106	—	106	106	106	30	30	30	30	30	30
Max	7.19	8.05	7.80	7.90	30.90	30.73	—	31.20	1254.6	1018.2	0.15	1.20	0.07	0.08	2.95	6.15
Min	4.83	5.24	7.20	7.30	28.70	29.42	—	28.4	839.5	874.9	0.05	0.50	< 0.05	< 0.05	1.80	2.55
**TC**_**2**_																
Mean	6.68	7.55	7.51	7.05	30.17	30.24	—	30.81	810.8	932.3	0.13	0.37	< 0.05	< 0.05	1.45	5.15
SD	1.06	2.29	1.64	1.02	4.95	3.67	—	5.79	17.4	19.7	0.02	0.07	0.00	0.00	0.04	1.51
*N*	106	106	106	106	106	106	—	106	106	106	30	30	30	30	30	30
Max	7.94	8.65	7.40	7.10	30.80	30.50	—	31.30	1157.3	1194.6	0.80	0.90	0.05	0.09	2.20	7.05
Min	4.03	5.48	7.00	7.00	30.10	30.00	—	29.50	865.1	913.8	0.05	0.20	< 0.05	< 0.05	1.05	3.40
**TC**_**3**_																
Mean	6.54	7.27	7.21	7.04	30.25	30.81	—	30.20	871.5	839.1	0.19	1.03	< 0.05	< 0.05	1.15	5.10
SD	1.74	2.42	1.73	1.22	4.77	4.02	—	5.42	16.7	21.3	0.03	0.64	0.00	0.00	0.00	1.36
*N*	106	106	106	106	106	106	—	106	106	106	30	30	30	30	30	30
Max	7.24	8.87	7.60	7.80	30.80	30.10	—	31.20	1260.4	1153.9	1.08	1.05	0.09	0.08	2.15	7.45
Min	3.71	4.64	7.00	6.90	29.50	28.60	—	30.10	753.5	842.0	0.45	0.87	< 0.05	< 0.05	0.95	3.95
**TC**_**4**_																
Mean	5.95	7.70	7.25	7.16	30.05	30.31	—	30.27	964.4	854.1	0.59	0.46	< 0.05	< 0.05	1.75	2.92
SD	2.06	2.29	1.86	1.34	3.27	3.12	—	3.86	18.5	15.9	0.05	0.03	0.00	0.00	0.03	0.03
*N*	106	106	106	106	106	106	—	106	106	106	30	30	30	30	30	30
Max	7.09	8.55	7.50	7.40	30.80	30.60	—	31.00	1163.6	1203.1	0.95	1.85	0.07	0.08	3.05	6.70
Min	2.43	5.59	7.01	7.49	30.10	29.40	—	29.80	857.5	840.3	0.30	0.45	< 0.05	< 0.05	1.20	2.05
**TC**_**5**_																
Mean	6.39	7.85	7.45	7.01	30.28	30.13	—	30.50	851.7	845.9	0.53	0.97	< 0.05	< 0.05	1.25	5.95
SD	1.13	2.09	1.94	1.42	2.59	3.78	—	4.52	21.4	14.9	0.06	0.05	0.00	0.00	0.08	1.30
*N*	106	106	106	106	106	106	—	106	106	106	30	30	30	30	30	30
Max	7.78	8.20	7.90	7.50	30.50	30.20	—	31.10	1182.7	1095.3	0.90	1.05	0.05	0.08	2.55	6.95
Min	5.95	5.04	7.20	7.26	29.20	28.90	—	30.30	790.5	832.8	0.50	0.75	< 0.05	< 0.05	0.95	2.75

Abbreviations: AP, aquaponic unit; HP, hydroponic unit; Max, maximum; Min, minimum; *N*, number of samples; SD, standard deviation.

Dissolved oxygen (DO) concentrations showed some variation between treatments, with mean values ranging from 5.95 mg/L (TC_4_ in HP) to 7.85 mg/L (TC_5_ in AP). Despite these fluctuations, DO levels were consistently above the minimum threshold reported as critical for tilapia and catfish survival, indicating that oxygen supply was not a limiting factor throughout the experiment. Water pH remained relatively stable across treatments, with values ranging between 7.04 ± 1.22 (TC_3_ in AP) and 7.85 ± 2.09 (TC_5_ in AP). The pH values generally fell within the optimal range (6.5–8.5) for freshwater aquaculture, ensuring good physiological conditions for both fish species and favorable nutrient availability for plants.

Water temperature values were also consistent, ranging from 30.05°C (TC_4_ in HP) to 30.81°C (TC_3_ in AP). Air temperature fluctuated between 28.6°C (TC_3_) and 31.3°C (TC_2_). These thermal conditions are considered suitable for tropical aquaponic systems and compatible with the growth requirements of Nile tilapia and African catfish, both of which are warm‐water species. Electrical conductivity (EC) exhibited moderate differences between treatments. In the hydroponic phase, values ranged from 810.8 (TC_2_) to 964.4 μS/cm (TC_4_), while in the aquaponic phase they varied between 765.3 (TC_1_) and 932.3 μS/cm (TC_2_). These values reflect nutrient availability in the system and remained within acceptable levels for combined fish and plant growth.

Regarding nitrogenous compounds, ammonium (NH_3_/NH_4_^+^) concentrations varied from 0.09 ± 0.03 mg/L (TC_1_ in HP) to 0.59 ± 0.05 mg/L (TC_4_ in HP). Nitrite (NO_2_^−^) levels remained below the detection limit (< 0.05 mg/L) in most cases, suggesting efficient nitrification processes within the aquaponic system. Nitrate (NO_3_^−^) concentrations, however, showed greater variation, ranging from 1.15 mg/L (TC_3_ in HP) to 5.95 mg/L (TC_5_ in AP). The presence of nitrates at these levels is expected in recirculating aquaponic systems and supports plant growth without reaching concentrations harmful to fish.

Taken together, these results confirm that water quality conditions were maintained within optimal or acceptable ranges during the entire experimental period. No critical deviations were observed that could negatively affect fish survival or growth or compromise the performance of the integrated aquaponic system.

#### 3.4.2 Effects of Environmental Conditions on Fish Performance

**FIGURE 5:**
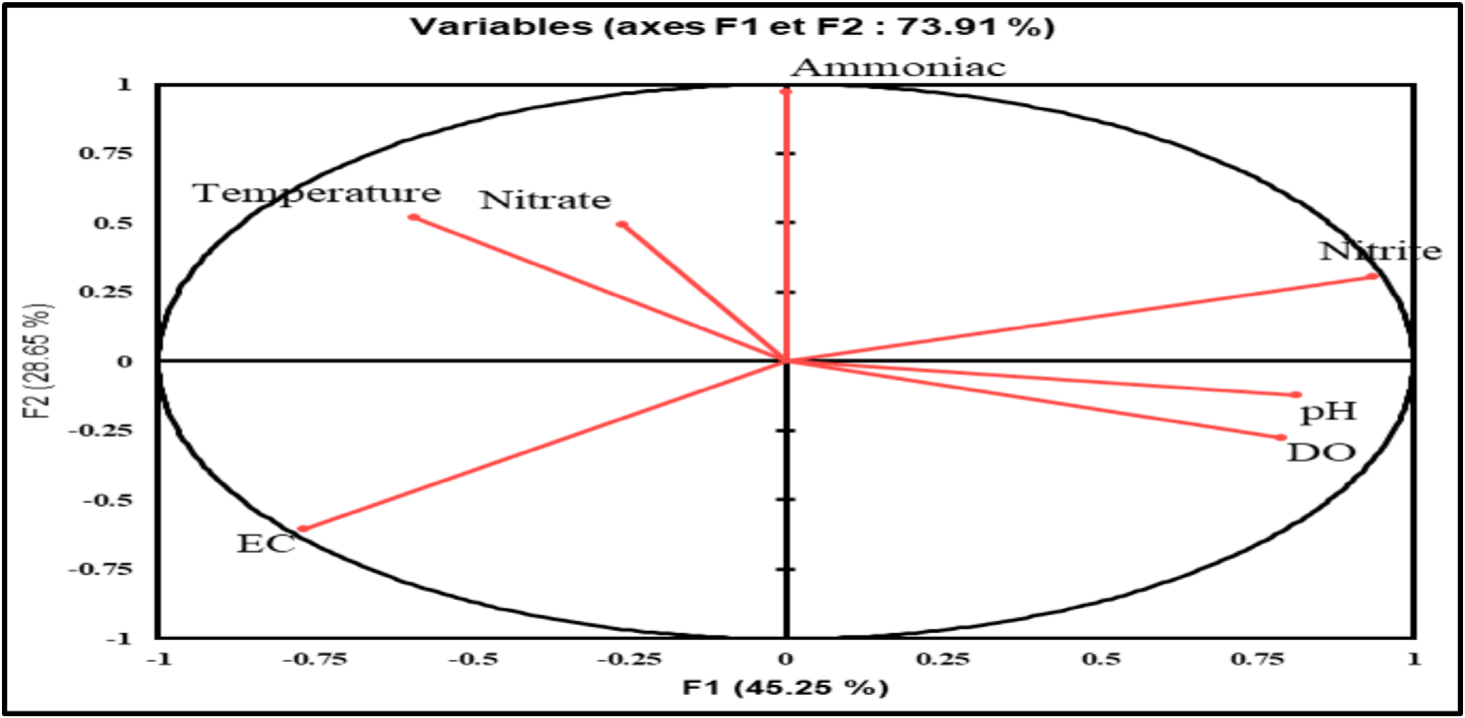
RAS water condition variables in terms of principal components analysis.

A principal component analysis was performed to assess the influence of environmental conditions (temperature, dissolved oxygen, pH, electroconductivity, ammonia, nitrite and nitrate) on fish growth. Figure 5 shows that ammonia, nitrite and temperature had the highest positive influence, while EC had a negative influence on fish. Nitrite, pH and OD appear to have a non‐significant effect on fish growth.

## 4 Discussion

Aquaponics represents an integrated approach combining aquaculture and horticulture, enabling simultaneous production of fish and plants (Rakocy et al. 2006). In this study, eggplant ( *Solanum melongena* ) was co‐cultivated with Nile tilapia ( *Oreochromis niloticus* ) and African catfish ( *Clarias gariepinus* ). Eggplant is an important fruit‐bearing vegetable in the Democratic Republic of Congo (DRC), widely used in the preparation of traditional sauces (Muzingu 2010). The production cycle lasted 106 days and was divided into two distinct phases: vegetative growth and fruit production. Across all treatments, the number of fruits harvested, fruit weight, and total fruit biomass did not differ significantly, suggesting that the availability of essential nutrients for eggplant growth and fruiting was relatively consistent in both qualitative and quantitative terms. This observation aligns with the findings of Somerville et al. (2014), who reported that when nutrient availability is maintained at similar levels, plant growth and yield in aquaponic systems are often comparable.

Despite this, the fruit yield per plant observed in this study was lower than that reported by Coulibaly et al. (2021), who recorded up to 80 fruits per plant. This discrepancy can be attributed to differences in cultivation systems, as Coulibaly et al. (2021) employed soil‐based cultivation, whereas this study utilized soilless aquaponics, in addition to variations in plant varieties. Moreover, the harvest interval likely contributed to reduced fruit production. Aglinglo et al. (2018) emphasized that frequent harvesting at short intervals (2–3 days) stimulates fruiting, whereas in this study, the harvest interval exceeded 5 days, potentially limiting total fruit yield.

Nile tilapia and African catfish are among the most widely cultured fish species in tropical Africa and urban areas of the DRC (Efole 2011; Lusasi et al. 2022). In polyculture systems, Nile tilapia typically serves as the primary species, with African catfish included to consume surplus tilapia fry, thereby controlling excessive reproduction (Elegbe et al. 2015). In certain systems, however, the combination aims to maximize simultaneous production of both species, leading to the adoption of various stocking ratios to optimize fish biomass productivity (Toko 2007).

Polyculture involving two or more species is a common practice in aquaculture. Previous studies include Toko et al. (2007) with *C. gariepinus* and *Heterobranchus longifilis* , Mondal et al. (2010) with *Anabas testudineus* and *O. niloticus* , and Nuwansi et al. (2017) with *Cyprinus carpio* and *Carassius auratus* . For Nile tilapia and African catfish specifically, pond trials were conducted by Middendorp (1995) and Elegbe et al. (2015), and aquaponics trials by Andriani et al. (2017), all of which reported favorable growth performance in associated species.

In the present study, Nile tilapia growth was not influenced by stocking ratios, whereas African catfish growth decreased with reduced numbers in the rearing tanks. Toko et al. (2007) reported that African catfish exhibit improved growth at higher stocking densities. Our observations corroborate this trend: despite maintaining a constant density of 10 kg·m^−3^, a decrease in the number of African catfish per tank led to reduced growth performance, with the effect most pronounced between treatments with the highest and lowest fish numbers. Overall, however, growth performance for both species remained acceptable across all treatments. Comparing Nile tilapia growth rates obtained here with those reported by Elegbe et al. (2015) indicates that co‐culture does not negatively affect tilapia growth in either pond or aquaponic systems. Survival rates in this study were exceptionally high, exceeding values reported by Licamele (2009) despite lower stocking densities, and consistent with Sabwa et al. (2021) for aquaponic systems.

Fish body composition, protein, lipid, ash, and moisture is a key indicator of nutritional quality (FAO 2004; Adeniyi et al. 2012). Fish from all treatments in this study displayed statistically similar nutrient profiles, with Nile tilapia exhibiting slightly higher protein content and African catfish showing elevated lipid levels. These results are consistent with the use of isoproteic, isoenergetic diets with identical nutrient compositions, confirming that differences in body composition are largely species‐specific, as previously observed by Adeniyi et al. (2012).

Heavy metals, natural constituents of freshwater environments, can also impact aquaculture systems. Elements such as Cu, Fe, and Zn are essential nutrients for plant growth (Neori et al. 2007; Nadya et al. 2016), while fish feeds may be contaminated with heavy metals through environmental sources, posing potential risks for fish and human consumers (Feldlite et al. 2008; Sarkar et al. 2021). Sarkar et al. (2021) and Salam et al. (2002) reported high concentrations of Pb, Cd, Cr, Cu, and Zn in some commercial feeds. In this study, seven heavy metals were analyzed. Consistent with previous reports (Museme et al. 2020; Salam et al. 1996), no Pb, Cd, or As were detected in either species, likely due to minimal accumulation and the probable absence of these metals in feed and water. Mercury (Hg) and manganese (Mn) concentrations were also below previously reported levels (Kouamenan et al. 2020; Catarina et al. 2011). Copper levels were well below safety thresholds, confirming the safety of fish consumption from this aquaponic system.

Water quality parameters are critical for optimizing aquaponic production, directly affecting fish metabolism, growth, welfare, and survival (Somerville et al. 2014). During the experiment, all measured variables were within acceptable ranges for the three biological species, supporting the observed growth performance and high survival rates.

## 5 Conclusion

Fish farming in the Democratic Republic of Congo primarily involves two main species: Nile Tilapia and African catfish, which are commonly reared in polyculture systems in earthen ponds. This study demonstrated the feasibility of simultaneously rearing these two species in the same aquaponic tank. Growth performance, survival rate, and nutritional body composition of Nile Tilapia did not significantly differ across treatments, indicating that its growth and survival are not adversely affected by co‐culture. However, African catfish showed a slight decline in growth performance with decreasing numbers in the rearing tanks.

The production of *Solanum melongena* (eggplant) was also unaffected by the fish co‐culture, with fruit number, weight, and biomass remaining statistically consistent among treatments.

Overall, this study contributes valuable insights to aquaponics research and presents a promising sustainable production approach for Nile Tilapia and African catfish in the Democratic Republic of Congo.

## Author Contributions

**Jude Kifufu Gafidji Fidji:** conceptualization, funding acquisition, writing – original draft, writing – review and editing, methodology, formal analysis. **Réné Wamene Baranger:** methodology. **Gaétan Kalala Bolokango:** validation, supervision. **Patrick Mafwila Kinkela:** visualization, data curation. **Honoré Kiatoko Mangeye:** validation.

## Data Availability

The data supporting the findings of this study are available from the corresponding author upon reasonable request.
